# Multi-Omics Identification of *Fos* as a Central Regulator in Skeletal Muscle Adaptation to Long-Term Aerobic Exercise

**DOI:** 10.3390/biology14060596

**Published:** 2025-05-24

**Authors:** Chaoyang Li, Xinyuan Zhu, Yi Yan

**Affiliations:** 1 Department of Sport Biochemistry, Sport Science School, Beijing Sport University, Beijing 100084, China; lichaoyang@bsu.edu.cn; 2 China Institute of Sport Science, Beijing 100061, China; zhuxinyuan@ciss.cn; 3 Laboratory of Sports Stress and Adaptation of General Administration of Sport, Beijing Sport University, Beijing 100084, China

**Keywords:** long-term aerobic exercise, skeletal muscle, *Fos*, *Tnfrsf12a*, RNA-seq, scRNA-seq, machine learning

## Abstract

Skeletal muscle health and function are closely linked to long-term aerobic exercise, particularly in enhancing muscle metabolism and regulating gene expression. Regular endurance training can significantly ameliorate metabolic dysfunction and prevent chronic diseases. However, the precise molecular mechanisms underlying skeletal muscle adaptations to long-term aerobic exercise require further clarification. To address this, we integrated transcriptomic and single-cell omics datasets from multiple long-term aerobic exercise models retrieved from the GEO database. After merging and batch correction, differential expression analysis identified 204 DEGs, including 110 upregulated and 94 downregulated genes. Key feature genes were screened using Lasso regression, SVM-RFE, and Random Forest machine learning algorithms, validated by RT-qPCR, and refined through PPI network analysis. Among them, *Fos* and *Tnfrsf12a* were significantly downregulated following long-term aerobic exercise. Notably, *Fos* exhibited a more pronounced decrease than *Tnfrsf12a*, and was strongly associated with inflammation and muscle regeneration. PPI network analysis indicated that *Fos* interacted with genes such as *Casp3*, *Egr1*, *Aft3, Hspa5*, *Src*, and *Igf2*. GO, KEGG, and GSEA enrichment analyses revealed that *Fos* is involved in skeletal muscle differentiation, tissue remodeling, and the NF-κB inflammatory pathway. ssGSEA analysis further showed that samples with low *Fos* expression had significantly elevated Th1/Th2 and Treg cell infiltration. Single-cell analysis confirmed preferential *Fos* expression in muscle fiber/adipocyte progenitors, satellite cells, and tenocytes, all critical for myogenesis. In summary, our findings suggest that long-term aerobic exercise downregulates *Fos*, potentially alleviating inflammation and enhancing satellite cell-mediated muscle regeneration. *Fos* may serve as a central regulator of skeletal muscle remodeling during long-term aerobic exercise.

## Simple Summary

Long-term aerobic exercise improves skeletal muscle health by promoting metabolism and gene regulation. In this study, we analyzed transcriptomic and single-cell data from multiple long-term aerobic exercise models to uncover key genes involved in muscle adaptation. Through machine learning and experimental validation, we identified *Fos* as a central regulator. *Fos* was downregulated after long-term exercise, which may promote muscle cell differentiation and reduce inflammation. These findings reveal new insights into how regular aerobic exercise reshapes skeletal muscle at the molecular level.

## 1. Introduction

Unhealthy lifestyles and behavioral habits have led to a significant increase in the incidence of various chronic diseases, placing a heavy economic burden on global healthcare systems [1]. Physical inactivity is a major contributor to numerous chronic diseases and is directly responsible for a considerable number of deaths. However, it is a modifiable risk factor that can be effectively mitigated through appropriate interventions [2]. Given its central role in metabolism and overall health, skeletal muscle is particularly susceptible to the detrimental effects of physical inactivity. In the human body, skeletal muscle is the most abundant tissue, accounting for approximately 30% to 40% of total body weight in healthy adults [3]. As a highly differentiated striated muscle tissue, skeletal muscle exhibits remarkable plasticity and responds significantly to both anabolic and catabolic stimuli. Its primary functions extend beyond active contraction and relaxation to supporting movement and maintaining posture; it also regulates various physiological processes by secreting a large number of myokines [4]. Physical inactivity is strongly associated with skeletal muscle-related disorders, including skeletal muscle insulin resistance [5], sarcopenia [6], and intramuscular fat infiltration [7].

Exercise rapidly induces differential expression of genes involved in immediate early responses, myogenic regulatory factors, carbohydrate and lipid metabolism, as well as mitochondrial biogenesis and oxidative phosphorylation [8]. These adaptations help in maintaining or enhancing skeletal muscle function. Moreover, exercise improves skeletal muscle mass and metabolic function by enhancing protein synthesis [9], increasing muscle mass [10], promoting amino acid synthesis [11], and improving insulin sensitivity [12]. These beneficial responses are primarily driven by metabolic enhancement and molecular remodeling processes in skeletal muscle during and after exercise [8].

To date, although numerous studies have investigated molecular responses to aerobic exercise, including AMPK, PGC-1α, mTOR, and others [13,14,15], the specific mechanisms governing skeletal muscle adaptation to long-term aerobic exercise remain to be fully elucidated. In recent years, with the rapid advancements in exercise physiology and molecular biology, transcriptomics has emerged as a powerful tool for elucidating complex gene expression patterns in biological systems [13]. In light of this, the present study integrates transcriptomic datasets from multiple GEO databases on long-term aerobic exercise models and employs bioinformatics approaches to investigate the biological functions of skeletal muscle regulation. Furthermore, machine learning and protein–protein interaction network construction are used to identify potential key genes. Finally, we systematically review existing single-cell transcriptomic datasets related to aerobic exercise to explore the expression characteristics of key regulatory factors in different skeletal muscle cell subpopulations. By elucidating the specific biological processes induced by long-term aerobic exercise in skeletal muscle, this study aims to provide novel targets for the prevention and treatment of skeletal muscle-related diseases and to advance theoretical understanding in support of exercise-based therapeutic strategies.

## 2. Materials and Methods

**Figure 1:**
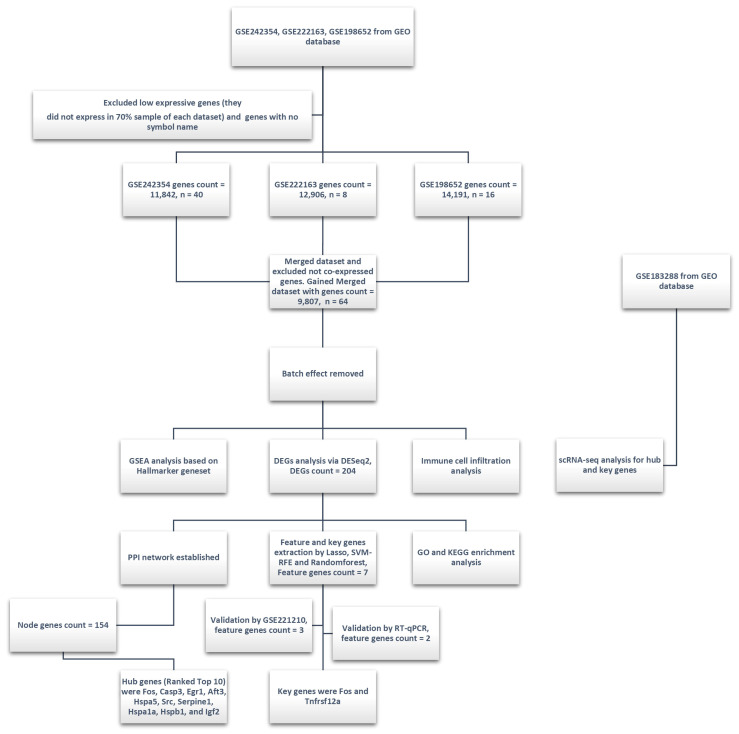
Experimental design and analysis workflow of this study. Three GEO datasets (GSE242354, GSE222163, and GSE198652) were integrated, with low-expression genes (expressed in less than 70% of samples per dataset) and genes without official symbols filtered out during preprocessing. The merged dataset (9807 genes, 64 samples) underwent batch effect removal. Subsequent analyses included GSEA based on the Hallmarker gene set, differential expression genes analysis (DESeq_2_, identifying 204 DEGs), immune cell infiltration analysis, and key feature gene selection using the Lasso, SVM-RFE, and Random Forest methods (resulting in 7 key genes). Key genes (*Fos* and *Tnfrsf12a*) were validated using the GSE221210 dataset and RT-qPCR. PPI network analysis identified 154 node genes, with hub genes ranked. GO and KEGG enrichment analyses DEGs provided insights into their potential biological functions and pathways. Additionally, single-cell transcriptomics analysis (scRNA-seq) was conducted using the GSE183288 dataset to further investigate hub and key genes. The scRNA-seq analysis helped in exploring the expression patterns of these genes across different cell populations.

An overview of the methodological framework in this study is presented in Figure 1.

### 2.1. Data Download and Batch Effect Removal

The raw count expression matrices of RNA sequencing data were downloaded from the GSE242354, GSE222163, and GSE198652 datasets in the GEO database. Using R v4.4.1, skeletal muscle samples were extracted, and gene expression data were merged by gene symbol using the merge_eset function. Genes that were not expressed in any of the datasets were excluded from further analysis. To correct for batch effects among datasets with or without long-term aerobic exercise intervention, the ComBat_seq function from the SVA package was applied. Principal component analysis (PCA) was performed to assess differences between datasets before and after batch correction. Following batch correction, a unified RNA-Seq expression matrix of skeletal muscle samples was obtained for downstream analyses. In cases where multiple probe sequences were mapped to the same gene, the probe with the highest expression value was retained to represent the gene. Specific characteristics of the included datasets are summarized in Table 1.

### 2.2. Differential Expression Genes and Clustering Analysis

First, the filterByExpr function from the edgeR package was used to filter low-expression genes to improve the statistical power of differential expression analysis. Subsequently, the DESeq_2_ algorithm in R v4.4.1 was applied to the count values of the total expression matrix for differentially expressed genes (DEGs) analysis. The thresholds for identifying DEGs were set as: log_2_ fold change (log_2_FC) > mean(log_2_FC) + 2 × SD(log_2_FC) and adj.P.Val < 0.05. Genes with negative log_2_FC values were defined as downregulated, while those with positive log_2_FC values were considered upregulated. A volcano plot was generated to visualize differential gene expression after long-term exercise intervention. The top 80 upregulated and downregulated genes were selected based on ascending adj.P.Val, and hierarchical clustering was performed using a heatmap function.

### 2.3. Feature Genes Extraction and Validation of the Training Set Based on Machine Learning Algorithms

This study employed three machine learning algorithms to identify feature genes following prolonged exercise. Initially, the original count expression matrix of DEGs was standardized using the Z-score method via the Scale function and served as the training dataset for subsequent machine learning applications. To ensure reproducibility, a fixed random seed was set for the Lasso regression analysis, which was performed using the glmnet package. The Lasso regression curve was plotted, followed by a ten-fold cross-validation that generated a cross-validation curve based on the lambda values.

Next, we utilized support vector machine–recursive feature elimination (SVM-RFE) to select feature genes post long-term aerobic exercise intervention. By loading the SVM-RFE algorithm, the expression matrix of DEGs was designated as the training dataset for SVM-RFE. A five-fold cross-validation was implemented for feature extraction, and the top 40 feature genes were selected for SVM-RFE model construction, with an error rate curve based on the SVM-RFE algorithm being plotted.

We then constructed a Random Forest model on the training dataset using the randomForest package, ensuring reproducibility by setting a fixed random seed for the Random Forest model. A total of 64 samples were randomly selected with replacement from the training dataset, and 14 feature genes were randomly chosen from all feature genes for decision splitting, resulting in the generation of 200 classical decision trees. The top 20 ranked genes based on the MeanDecreaseGini from the Random Forest model were selected as the predicted feature genes.

Finally, we took the intersection of the results obtained from Lasso regression, SVM-RFE, and Random Forest feature extraction, counted the number of intersecting genes, and visualized the gene extraction results using Venn diagrams and UpSet plots. The intersecting genes were further validated in an external validation set, GSE221210 (Protocol for long-term aerobic training: start at 10 m/min, increase 0.5 m/min daily to 19.5 m/min, 1 h/day for 4 weeks, 5° incline. For acute exercise: 5 min at 5 m/min, then 5 min at 8 m/min, followed by 2 m/min increments every 15 min until exhaustion, 5° incline), in which those exhibiting trends consistent with the training set and showing marginally significant differences in adjusted *p*-values (adj.P.Val < 0.15) were considered successfully validated. Additionally, a single-gene ROC curve analysis was performed on the selected genes. The expression of *Fos* at 0, 4, 6, and 8 h following acute exercise (from GSE221210) was analyzed using the Wilcoxon test to evaluate its temporal changes.

### 2.4. Sample Collection and RT-qPCR

To ensure the stability of the results, this study validated the feature genes selected by machine learning using RT-qPCR, thus guaranteeing the reproducibility of the findings. First, a long-term exercise model was established using mice. Sixteen C57BL/6J mice were purchased and divided into two groups: an 8-week control group (control, *n* = 8) and a long-term exercise intervention group (exercise, *n* = 8). After an adaptive feeding period, all mice underwent 5 days of treadmill acclimatization training, with each training session lasting 10 min at a speed of 9 m/min and a slope of 0°. Subsequently, each mouse in the exercise group was assigned to a relatively independent treadmill for a formal training period of 8 weeks. The exercise training intervention occurred from Monday to Friday at 6 p.m., with weekends reserved for rest. The treadmill speed and training duration schedule during the training period were as follows: each training session lasted for 60 min, starting with a warm-up at 9 m/min for 5 min, followed by the main training phase at a speed of 12–15 m/min (75–80% VO_2max_) for 50 min, and concluding with a cool-down at 5 m/min for 5 min. The main training speed increased from 12 m/min by 1 m/min every two weeks until it reached 15 m/min by the sixth week, after which it remained constant. The treadmill slope was set to 0°, while the control group of mice remained stationary on another treadmill for 60 min. Twelve hours after completing the 8-week aerobic exercise intervention, all mice were anesthetized with isoflurane and euthanized via cervical dislocation. Bilateral gastrocnemius muscles were excised, rapidly frozen in liquid nitrogen, and stored at −80 °C for subsequent analyses.

Total RNA was extracted from the bilateral gastrocnemius muscles using the TRIzol–chloroform–isopropanol method. Bilateral gastrocnemius muscles tissues from each group of mice were placed in a glass homogenizer, and 1 mL of TRIzol reagent (Invitrogen, Carlsbad, CA, USA) was added for lysis. The samples were homogenized thoroughly for 5 min to ensure complete dissolution of the protein–RNA complex. Next, 0.2 mL of chloroform was added, the cap was closed, and the mixture was vigorously shaken for 10 s, followed by an incubation period of 2–3 min. The samples were then centrifuged at 12,000× *g* for 15 min at 4 °C. After centrifugation, the upper aqueous phase was carefully transferred to a new EP tube for RNA extraction, maintaining a 45° angle with the tube. To precipitate RNA, 0.5 mL of isopropanol was added to the aqueous phase, gently mixed, and incubated at 4 °C for 10 min. Following this, the samples were centrifuged at 12,000× *g* for 10 min at 4 °C. The supernatant was completely discarded, and the RNA pellet was retained. Residual liquid was removed with a pipette. The RNA pellet was resuspended in 1 mL of 75% ethanol. After briefly vortexing the EP tube, the samples were centrifuged at 7500× *g* for 5 min at 4 °C. The supernatant again was completely discarded, and the RNA pellet was retained. Any residual liquid was removed with a pipette. The cap was opened, and the RNA pellet was air-dried at room temperature for 5–10 min. Finally, 20–50 μL of RNase-free water was added to resuspend the RNA pellet, which was incubated in a 55 °C heating block for 10 min to ensure complete dissolution. The concentration of total RNA in each sample was measured using a micro-volume spectrophotometer, followed by normalization of the concentrations.

Subsequently, a reverse-transcription system of 20 μL was constructed to synthesize cDNA. The nucleotide sequences of the target genes were obtained from the NCBI and Primer Bank databases to design forward and reverse primers. The specific nucleotide sequences of the forward and reverse primers for each target gene are listed below (Table 2). After obtaining the primers, each reaction well received 5 μL of SYBR Green fluorescent dye, 0.2 μL of 10 μM forward and reverse primers, 1 μL of cDNA, and 3.6 μL of RNase-free water to construct a 10 μL RT-qPCR system. The following thermal cycling conditions were set: pre-denaturation at 95 °C for 30 s, denaturation at 95 °C for 15 s, annealing at 60 °C for 30 s, and extension at 72 °C for 30 s, with fluorescence signal collection immediately after the extension phase. A total of 40 cycles of denaturation, annealing, and extension were performed to complete the RT-qPCR. Finally, the relative expression levels of the target genes were calculated using the formula 2^−△△Ct^ based on the Ct values of each reaction well. One-way ANOVA was employed to analyze the relative expression levels of mRNA, with *p* < 0.05 set as the threshold for statistical significance.

### 2.5. Protein–Protein Interaction (PPI) Analysis and Hub Gene Extraction Based on Cytoscape

DEGs were analyzed for PPI using the STRINGdb package, utilizing version 12.0 of the STRING database. The threshold for the protein interaction score was set at 400. After deriving the PPI interaction network and exporting the interaction information among DEGs, the analysis was conducted using the cytoHubba plugin within Cytoscape v3.10.2 software. The maximum clique centrality algorithm was employed to identify hub genes that had centrality within the largest clique, thus selecting the top 10 hub genes based on their scores.

### 2.6. GO, KEGG, and Gene Set Enrichment Analysis (GSEA)

GO enrichment analysis was performed using the enrichGO function from the clusterProfiler package, with the *p*-value correction method set to false discovery rate (FDR) and both the *p*-value and *q*-value thresholds set at 0.05. The results of the GO enrichment analysis were categorized into biological processes (BPs), cellular components (CCs), and molecular functions (MFs), with visualizations provided through bubble plots and chord diagrams. The enrichment factor (EF) was calculated using the formula GeneRatio/BgRatio.

For KEGG analysis, the bitr function in RStudio was used to convert the symbols of DEGs to their corresponding ENTREZ IDs. Genes that could not be mapped to the corresponding ENTREZ IDs were excluded from subsequent KEGG analysis. The enrichKEGG function from the clusterProfiler package was then utilized for KEGG enrichment analysis, with the *p*-value correction method also set to FDR and the *p*-value and *q*-value thresholds remaining at 0.05. Similar visualizations using bubble plots and chord diagrams were employed to present the KEGG enrichment results. The EF was calculated using the same formula.

For GSEA analysis, the bitr function was used to convert the symbols in DEGs to ENSEMBL format, and the log_2_FC values of the DEGs were sorted in descending order. The msigdbr package was utilized to obtain gene sets and species information, with the hallmark gene set (H) designated as the gene set for the GSEA analysis in this study. The clusterProfiler package was then employed to perform GSEA analysis on the total log_2_FC expression data from DESeq_2_, setting a fixed random seed. The minimum gene number threshold for the gene set was set at 5 and the maximum at 5000, with an adjusted *p*-value threshold of 0.05. The *p*-value correction method was set to FDR, and the number of simple permutations was set to 5000. A change was considered statistically significant for enriched pathways when −log_10_(P.Val) > 1.3. The results were visualized using bubble plots, bar charts, and GSEA enrichment plots.

### 2.7. Immune Cell Infiltration Analysis

Immune cell infiltration analysis was conducted using the single sample gene set enrichment analysis (ssGSEA) algorithm on the original expression matrix. An annotation file containing marker genes for 16 types of immune cells (LM16.csv) was downloaded from previous literature [14]. The gsva package’s gsvaParam function was utilized to analyze the original expression matrix and score the 16 immune cell-related markers for each sample, specifying the distribution type for the samples as “Poisson”. The expression matrix underwent Z-score transformation. If the expression of *Fos* in a sample was greater than 0, it was classified as the *Fos* high-expression group; if it was less than 0, it was classified as the *Fos* low-expression group. Box plots were generated to visualize immune infiltration based on *Fos* expression levels. The Kruskal–Wallis test was employed for statistical analysis of the differences in immune cell expression between groups, with a significance level defined as *p* < 0.05. Pearson correlation analysis was conducted to assess the associations among immune cells and between genes and immune cells.

### 2.8. Single-Cell Transcriptomics Analysis

The sparse matrix of skeletal muscle single-cell transcriptomics was downloaded from GSE183288. The UMAP method from the Seurat R package was utilized for dimensionality reduction of the single-cell data, categorizing the skeletal muscle cell types into 16 cell types and 41 cellular subpopulations based on the annotation file. UMAP dimensionality reduction was also performed according to the intervention methods, comparing the sedentary control group (SC) and aerobic exercise intervention group (TC), as well as key genes. Finally, the Kruskal–Wallis test was employed for statistical analysis of the differences in the proportions of various cellular subpopulations between groups, with the significance level defined as *p* < 0.05.

## 3. Results

### 3.1. DEGs Analysis Based on DESeq_2_ Algorithm

**Figure 2:**
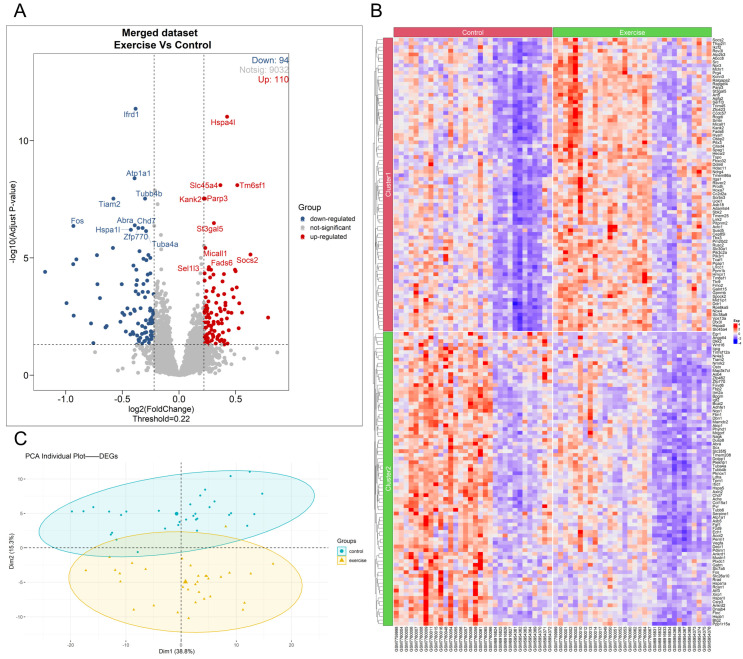
Analysis results of skeletal muscle DEGs after long-term aerobic exercise intervention via DESeq_2_ Algorithm. The volcano plot of DEGs in the long-term aerobic exercise group compared with the sedentary control group (**A**). The expression heatmap of DEGs (**B**). The PCA plot of DEGs after dimensionality reduction (**C**).

After filtering out low-expression genes, the merged expression matrix included a total of 9236 genes for DEGs analysis. The DESeq_2_ algorithm was used to perform DEGs analysis on the count expression matrix of these genes, with thresholds set at −log_10_(adj.P.Val) > 1.3 and |Log_2_FC| > 0.22 to identify genes with significant differences. The analysis revealed a total of 204 DEGs in skeletal muscle following long-term aerobic exercise, including 110 upregulated and 94 downregulated genes (Figure 2A). Significantly upregulated genes included *Hspa4l*, *Tm6sf1*, *Slc45a4*, *Parp3*, and *Kank2*, while downregulated genes included *Ifrd1*, *Atp1a1*, *Tiam2*, *Tubb4b*, and *Fos* (Figure 2A). The expression patterns of specific DEGs across samples can be seen in the clustering heatmap (Figure 2B), and the PCA of these DEGs between the two groups is shown in Figure 2C.

### 3.2. Machine Learning for Key Gene Extraction and Validation with Verification Set and RT-qPCR

**Figure 3:**
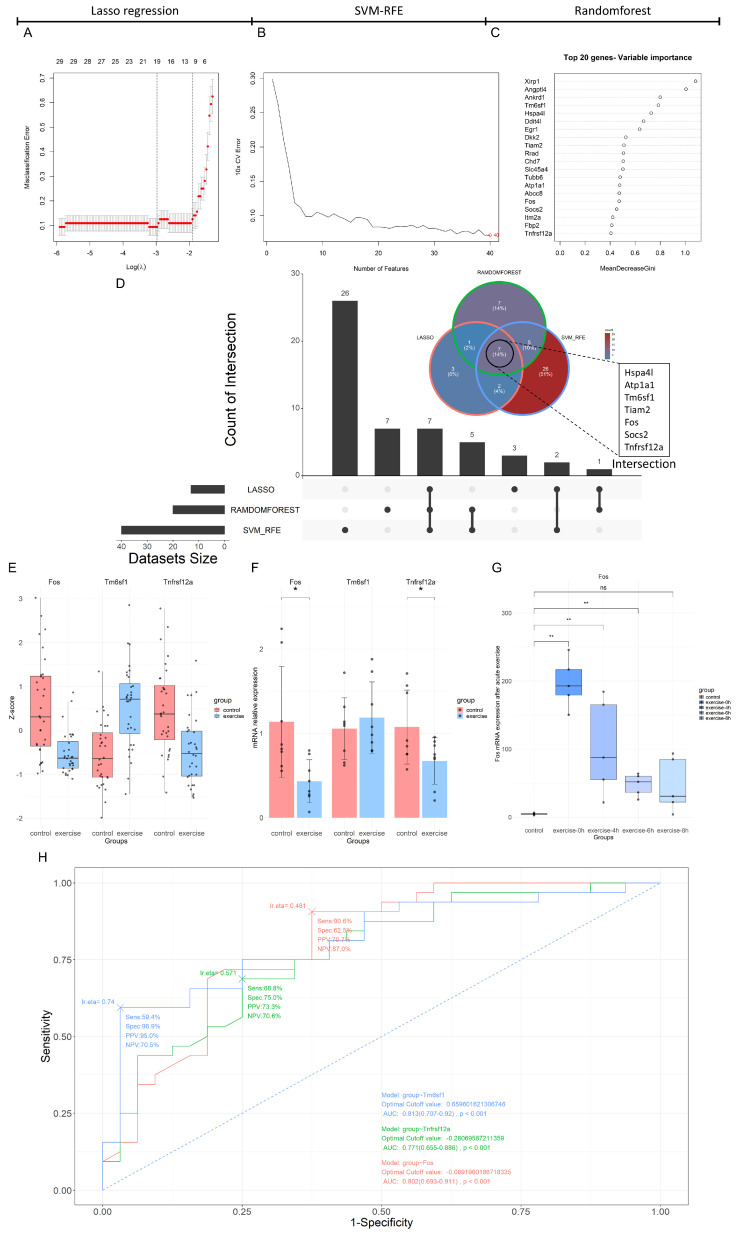
Multi-method feature extraction and validation results based on Lasso regression, SVM-RFE, and Random Forest. The misclassification error of the Lasso regression model across varying the Log(λ) value (**A**). The cross-validation error curve of the SVM-RFE model with respect to the number of features, highlighting the optimal number of features marked at the end of the curve (**B**). The top 20 genes ranked by variable importance in the Random Forest model, sorted by the Gini coefficient (**C**). The Venn and Upset plots of the feature genes selected by the Lasso regression, SVM-RFE, and Random Forest methods (**D**). The bar plot of *Fos*, *Tnfrsf12a*, and *Tm6sf1* expression in the merged expression matrix (**E**). The RT-qPCR results for *Fos*, *Tm6sf1*, and *Tnfrsf12a* genes in an 8-week aerobic exercise intervention model, where * indicates a significance level of *p* < 0.05 (**F**). The expression of the *Fos* gene in the acute exercise validation set, where ** indicates a significance level of *p <* 0.01, and “ns” indicates no significant difference (**G**). The ROC curves for *Fos*, *Tnfrsf12a*, and *Tm6sf1* (**H**).

In this study, three machine learning algorithms—Lasso regression, SVM-RFE, and the Random Forest model—were employed to further select key feature genes from the 204 DEGs. The Lasso regression and SVM-RFE methods selected 13 and 40 feature genes, respectively (Figure 3A,B). The top 20 feature genes ranked by the Gini index in the Random Forest model included *XirP1*, *Angptl4*, *Ankrd1*, and *Tm6sf1* (Figure 3C). The intersection of the feature genes selected by all three methods consisted of seven genes: *Hspa4l*, *Atp1a1*, *Tm6sf1*, *Tiam2*, *Fos*, *Socs2*, and *Tnfrsf12a* (Figure 3D). Subsequently, DEG analysis was performed using the validation set GSE221210, revealing that three of these seven feature genes showed consistent trends with the machine learning extraction results, and adj.P.Val < 0.15 (Table 3). ROC curves were used to evaluate the binary classification performance of these three feature genes, showing that the area under the curve (AUC) for each gene was >0.7 with *p* < 0.001, indicating high classification ability (Figure 3H). Therefore, these three feature genes were included for further RT-qPCR validation. The expression of the three feature genes in the merged dataset is shown in Figure 3E.

As shown in Figure 3F, the RT-qPCR results indicated that the intergroup difference in *Tm6sf1* mRNA expression was not statistically significant [*p* = 0.515, η^2^ = 0.031, F(1, 14) = 0.447], while significant differences were observed for *Fos* mRNA expression (*p* = 0.013, η^2^ = 0.346, F(1, 14) = 7.999) and *Tnfrsf12a* mRNA expression (*p* = 0.045, η^2^ = 0.256, F(1, 14) = 4.828). The trends for *Fos* and *Tnfrsf12a* mRNA were consistent with those observed in the training set, with both *Fos* and *Tnfrsf12a* mRNA being significantly downregulated after eight weeks of aerobic exercise intervention (Figure 3F).

Using acute exercise model data from the validation set, it was found that, in contrast with long-term aerobic exercise, *Fos* expression was significantly upregulated at 0, 4, and 6 h post-acute exercise (*p* < 0.01, Figure 3G), indicating differential regulation of *Fos* between acute and long-term aerobic exercise.

### 3.3. PPI Network Analysis and Extraction of Hub Genes Related to Fos Gene

**Figure 4:**
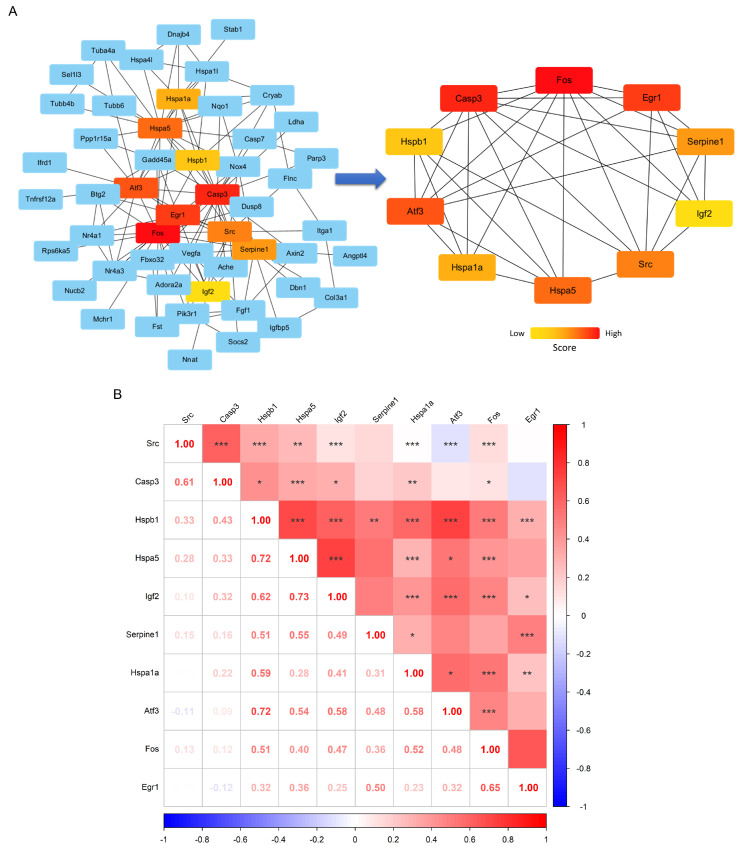
Construction of key gene networks and correlation analysis. The subnetwork of DEGs based on the STRING database (**left**) and the high-score core gene network (**right**), with colors ranging from red to yellow representing different gene scores (**A**). The expression correlation heatmap of key interacting genes (**B**), where the numbers indicate the correlation values, and *, **, *** represent significance levels of *p* < 0.05, *p* < 0.01, and *p* < 0.001, respectively.

Subsequently, PPI network analysis was performed on the DEGs, with a detailed diagram available in the Attachment. A total of 154 node genes and 285 interaction edges were identified, forming a common PPI subnetwork (Figure 4A). This PPI subnetwork was then analyzed using Cytoscape v3.10.2 software to screen for hub genes, with the top 10 hub genes being *Fos*, *Casp3*, *Egr1*, *Aft3*, *Hspa5*, *Src*, *Serpine1*, *Hspa1a*, *Hspb1*, and *Igf2* (Figure 4B). Among these, *Fos* overlapped with the key genes identified by the three machine learning algorithms and the positive results from RT-qPCR validation. Furthermore, correlation analysis was performed among the hub genes, revealing that *Fos* was significantly positively correlated with all the other hub genes except for *Serpine1* (Figure 4B). This suggests that *Fos* may be a key gene mediating the biological processes of skeletal muscle induced by long-term aerobic exercise.

### 3.4. GO, KEGG, and GSEA Enrichment Analysis

**Figure 5:**
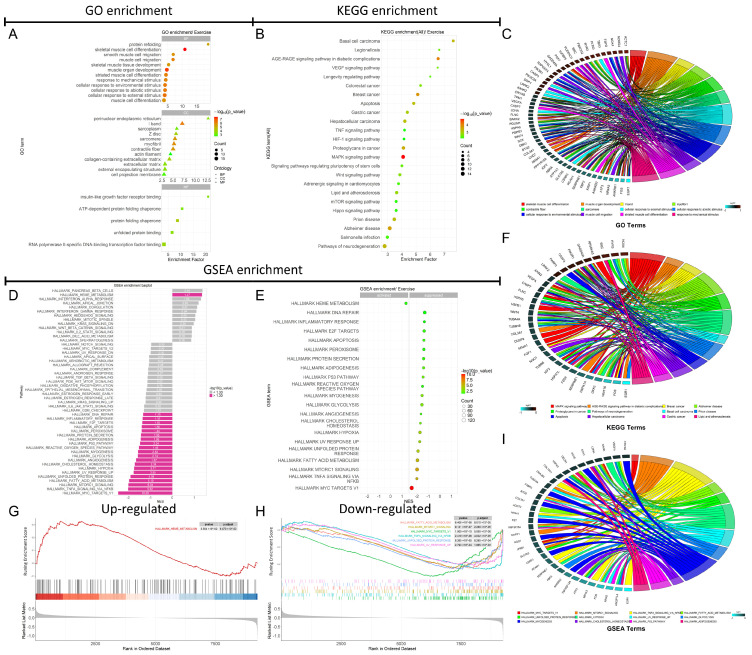
GO and KEGG functional enrichment analysis results and GSEA results based on the hallmark gene set. The GO enrichment bubble chart (**A**). The KEGG enrichment bubble chart (**B**). The gene chord diagram involved in the GO enrichment process (**C**). The gene chord diagram associated with the KEGG enrichment pathways (**F**). The GSEA analysis bar chart for the 50 enrichment items in the Hallmark gene set (**D**). The bubble chart for the analysis of 21 significantly enriched items in the Hallmark gene set (**E**). The GSEA plot for the top 1 down-regulated pathway based on the NES score (**G**). The GSEA plots for the top 6 downregulated pathways based on the NES score (**H**). The gene chord diagram associated with GSEA enrichment pathways (**I**).

In the GO enrichment analysis, significant terms with *p* < 0.05 and the top 12 enrichment terms across BP, CC, and MF were highlighted. The GO enrichment results (Figure 5A) indicated that, in BP, DEGs were significantly enriched in muscle cell development and differentiation, protein folding, response to mechanical stimulus, and response to external stimulus. In CC, DEGs were notably enriched in components such as the sarcoplasmic reticulum, sarcomere, myofibrils, I band, Z line, actin, extracellular matrix, and other structural components. In MF, significant enrichment was observed in functions like insulin-like growth factor receptor binding, protein folding chaperone activity, ATP-dependent protein folding, unfolded protein binding, and RNA polymerase II-specific DNA-binding transcription factor activity. The *Fos* gene was particularly enriched in GO terms related to skeletal muscle cell differentiation, muscle growth, response to external stimulus, and mechanical stimulus (Figure 5C). For details on other GO enrichment terms and associated DEGs, refer to Figure 5C.

The KEGG enrichment analysis showed that DEGs were significantly associated with pathways such as longevity regulation, lipid metabolism and atherosclerosis, pluripotency of stem cells, apoptosis, VEGF, HIF-1, TNF, MAPK, Wnt, mTOR, and Hippo signaling pathways (Figure 5B). The *Fos* gene was predominantly enriched in pathways related to MAPK signaling, breast cancer, apoptosis, and lipid metabolism/atherosclerosis (Figure 5F). For details on other KEGG enrichment terms and associated DEGs, refer to Figure 5F.

The GSEA enrichment analysis was performed using the Hallmark gene set on the 9236 genes analyzed with DESeq_2_, revealing significant enrichment in 21 out of 50 pathways within the Hallmark gene set (−log_10_(P.Val) > 1.3) (Figure 5D,E). Among these, 29 Hallmark pathways showed no significant enrichment (Figure 5D). There was one upregulated Hallmark pathway, which was heme metabolism, while 20 pathways were downregulated, including MYC targets V1, NFκB/TNF-α signaling, mTORC1 signaling, fatty acid metabolism, UV response, unfolded protein response, reactive oxygen species, p53 pathway, apoptosis, and inflammatory response pathways (Figure 5E). The top six GSEA enrichment scores and core gene sets, sorted by ascending *p*-value, are shown in Figure 5G,H. The top 12 enriched Hallmark pathways for DEGs are presented in Figure 5I. The *Fos* gene was significantly enriched in pathways such as NFκB/TNF-α signaling, UV response, p53 signaling, and fatty acid metabolism (Figure 5I). Notably, all these pathways were significantly downregulated according to the GSEA enrichment analysis (Figure 5D,E). This suggests that the downregulation of *Fos* may contribute the suppression of these pathways, potentially inhibiting inflammatory responses and apoptosis in skeletal muscle.

### 3.5. Immune Cell Infiltration Analysis by ssGSEA

**Figure 6:**
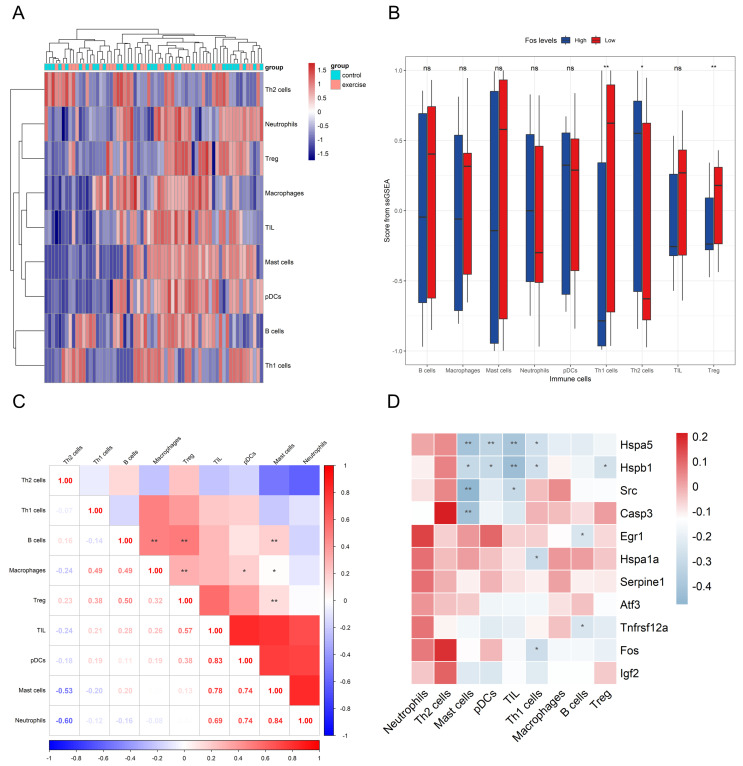
The impact of *Fos* in immune cell subpopulations. The inter-group expression heatmap of different immune cell subsets (**A**). Box plots comparing the expression levels in various immune cell subsets between the *Fos* high-expression and low-expression groups (**B**). The correlation heatmap among different immune cell subsets (**C**). The correlation heatmap between key genes and various immune cell subsets (**D**). In panels (**B**–**D**), * and ** represent significance levels of *p* < 0.05 and *p* < 0.01 respectively, and “ns” indicates no significant difference.

ssGSEA was conducted on the genes identified from machine learning and PPI network analysis to assess immune cell infiltration based on 16 immune cell markers, focusing on the role of the *Fos* gene in skeletal muscle inflammation. The results showed that, out of the 16 immune cell types, only 9 were expressed in the merged dataset. Among these, Th1 and Treg cells exhibited higher infiltration levels in the exercise group, while Th2 cell levels were significantly reduced, suggesting that long-term aerobic exercise may influence inflammation by modulating these immune cell levels (Figure 6A).

In skeletal muscle samples with low *Fos* expression, Th1 and Treg cell infiltration was increased, whereas Th2 cell infiltration was decreased, aligning with exercise group. Additionally, a significant positive correlation between high *Fos* expression and Th1 cell infiltration was found (*p* < 0.05), indicating that *Fos* may play a crucial role in regulating Th1 cell-mediated immune responses (Figure 6B). Correlation analysis revealed (Figure 6D) a statistically significant relationship between *Fos* and Th1 cells (r = −0.287, *p* < 0.05), while the correlation between *Fos* and Treg cells was near marginal significance (r = −0.196, *p* = 0.12). These findings suggest that *Fos* may regulate skeletal muscle immune and inflammatory responses by influencing the balance between Th1 and Th2 cells, as well as modulating Treg cell expression. The correlation heatmap among different immune cell subsets (Figure 6C) revealed distinct clustering patterns, suggesting potential interactions and co-regulation among these cell types.

### 3.6. Single-Cell Transcriptomic Analysis

**Figure 7:**
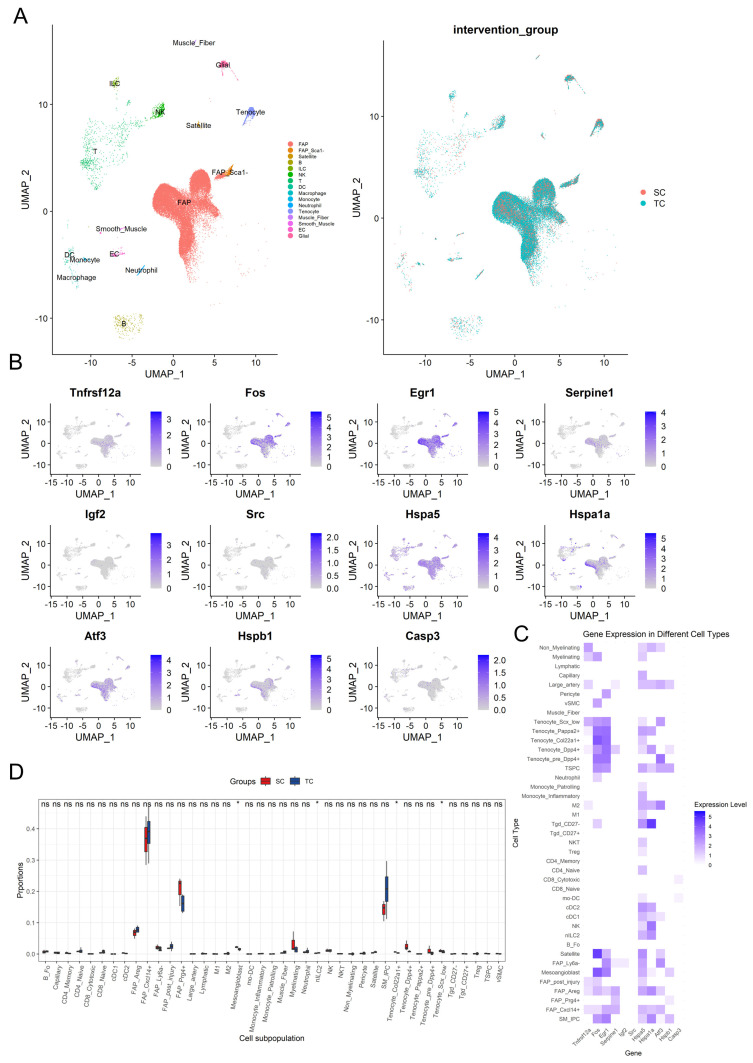
Key gene expression and distribution characteristics in different cell populations after aerobic exercise intervention. The UMAP dimensionality reduction plot of 16 cell types (**left**) and the group-wise UMAP plot (**right**) (**A**). The UMAP plot for key genes (**B**). The expression heatmap of key genes across 41 cell subsets (**C**). The proportion differences of various cell subsets in the SC and TC groups, with red and blue representing the sedentary control group (SC) and aerobic exercise intervention group (TC) respectively, where * represents significance levels of *p* < 0.05, and “ns” indicates no significant difference (**D**).

In this study, we analyzed skeletal muscle samples from the single-cell RNA sequencing data of GSE183288 to assess the impact of aerobic exercise intervention on the composition of various cell populations and the expression changes of key genes such as *Fos* in different cell groups. Figure 7A displays the cell population distribution based on UMAP dimensionality reduction. The left panel displays the distribution of various cell types in skeletal muscle tissue, which mainly includes fibro/adipogenic progenitors (FAPs), smooth muscle cells, myofibers, tenocytes, macrophages, neutrophils, satellite cells, and monocytes, totaling 16 distinct cell types (Figure 7A). Among these, FAPs constituted the dominant population across samples. Notably, distinct differences in cell composition were observed between the SC and TC. The right panel further reveals the differences between the intervention groups through UMAP analysis, indicating that the composition or distribution characteristics of the two groups were influenced by the aerobic exercise intervention (Figure 7A). Additionally, we analyzed the expression of multiple key genes in each cell type, focusing on the expression patterns of genes related to muscle regeneration and inflammation in different cells. The results showed that immediate early genes (IEGs) such as *Egr1* and *Fos* exhibited high expression levels in FAP and tenocyte cells, while *Fos* also showed substantial expression in satellite cells. *Egr1* also showed significant expression in smooth muscle (Figure 7B,C). The UMAP plots of gene expression suggest that these genes may play important roles in the different cell populations after intervention, further indicating the key regulatory role of FAP cells during aerobic exercise. By further annotating the 16 cell populations into specific subpopulations, we examined the expression patterns of key genes across different cell subpopulations. The results indicated that the 16 cell types in skeletal muscle were classified into a total of 41 subpopulations, with differential expression levels of genes such as *Fos* and *Igf2*, observed in FAP cells, satellite and mesoangioblast cells (Figure 7C). Importantly, FAPs and satellite exhibited elevated expression of multiple genes associated with muscle regeneration such as *Fos* and *Egr1*, reinforcing their essential role in the skeletal muscle repair process following long-term aerobic exercise.

To explore the differences in cell proportions between the SC and TC groups, we employed the Kruskal–Wallis test for intergroup analysis of the 41 cell subpopulations (Figure 7D). The analysis revealed significant changes in proportions within the Satellite, SM_IPC, and FAP subpopulations, with the TC group showing an increasing trend in Satellite (*p* = 0.289) and SM_IPC (*p* = 0.158) cell subpopulations. Furthermore, the proportion of other FAP subpopulations significantly increased in the TC group, except for FAP_Ly6a- (*p* (FAP_Ly6a−) = 0.077), although other FAP cell subpopulations did not show statistically significant differences (*p* (FAP_Cxcl14+) = 0.480, *p* (FAP_Prg4+) = 0.157, *p* (FAP_Areg) = 0.157, *p* (FAP_post_injury) = 0.289). Additionally, significant decreases in proportion were observed in the TC group for Mesoangioblasts (*p* = 0.034), Tenocyte_Col22a1+ (*p* = 0.034), and Tenocyte_Scx_low (*p* = 0.034), while nILC2 (*p* = 0.034) showed a significant increase. These results reveal that aerobic exercise intervention may influence skeletal muscle metabolism by regulating these cell types.

## 4. Discussion

In this study, we integrated and analyzed multiple multi-omics datasets of long-term aerobic exercise, employing machine learning algorithms to identify key genes. A series of enrichment and clustering analyses were conducted to elucidate the biological functions of *Fos* and *Tnfrsf12a*. Using machine learning methods, including Lasso regression, SVM-RFE, and Random Forest, combined with RT-qPCR experiment and validation datasets, we identified *Fos* and *Tnfrsf12a* as critical genes in the regulation of skeletal muscle metabolism during long-term aerobic exercise. Furthermore, our findings indicate that *Fos* is a key distinguishing gene between long-term aerobic exercise and acute exercise. Therefore, this study provides an in-depth discussion of the roles of *Fos* and *Tnfrsf12a* in the regulation of skeletal muscle under long-term aerobic exercise.

### 4.1. Tnfrsf12a’s Role in Exercise-Induced Modulation of Skeletal Muscle Function

*Tnfrsf12a*, a member of the tumor necrosis factor receptor superfamily, is also known as fibroblast growth factor-inducible 14 (Fn14). It is minimally expressed in normal tissues and under quiescent conditions [15]. Fn14 is the only known receptor for the tumor necrosis factor-like weak inducer of apoptosis (TWEAK), and together they form the TWEAK/Fn14 pathway, which is involved in critical biological processes such as cell proliferation, migration, differentiation, apoptosis, angiogenesis, and inflammatory responses [16]. Although the role of the TWEAK/Fn14 pathway in skeletal muscle has been widely studied, conflicting results have been reported, possibly due to variations in the expression levels of TWEAK and Fn14. Studies have shown that activation of the TWEAK/Fn14 pathway is highly associated with the pathogenesis of various acute and chronic muscle atrophy disorders [15]. Among these, the relationship between TWEAK and muscle atrophy is the most well-established. TWEAK induces skeletal muscle atrophy by inhibiting the phosphorylation of Akt kinase and its downstream targets, including GSK-3β, FOXO1, mTOR, and p70S6K, while also upregulating nuclear factor kappa B (NF-κB) expression. This leads to the suppression of the PI3K/Akt signaling pathway and the activation of both the ubiquitin-proteasome and NF-κB systems [17]. In contrast, studies have shown that primary myoblasts from Fn14 knockout mice exhibit reduced proliferative capacity and impaired myotube formation upon differentiation induction [18]. Additionally, subsequent studies have reported that Fn14 KO mice display diminished skeletal muscle regeneration following injury. These findings suggest that the Fn14 receptor may function independently of TWEAK, eliciting distinct biological responses that could involve as-yet unidentified ligands [19,20]. Fn14 mRNA expression is upregulated under acute and chronic pathological conditions, such as muscle denervation, cachexia, cancer, fasting, and diabetes. The diverse roles of the TWEAK/Fn14 pathway are determined by the relative levels of these two molecules. When both TWEAK and Fn14 are upregulated, TWEAK forms trimers that bind to the extracellular domain of Fn14, leading to the clustering of Fn14’s C-terminal region and triggering ligand-dependent activation. This activation mechanism is associated with chronic inflammation and pathological muscle atrophy [21]. Conversely, when TWEAK levels remain unchanged while Fn14 is upregulated or overexpressed, Fn14 undergoes ligand-independent activation through self-association of its C-terminal domain [22], a process typically observed in skeletal muscle following exercise [21].

#### Exercise-Induced Modulation of TWEAK/Fn14 Expression

Emerging evidence suggests that exercise differentially regulates TWEAK and its receptor Fn14 in skeletal muscle. Long-term aerobic exercise has been shown to reverse tumor-induced TWEAK upregulation and prevent muscle atrophy [23]. However, most studies indicate that exercise primarily affects Fn14 expression. Both acute and chronic resistance exercise significantly increased Fn14 mRNA and protein levels in human and mouse skeletal muscle, while TWEAK expression remained largely unchanged [24,25,26]. Similarly, physically active monozygotic twins exhibited higher Fn14 mRNA levels compared with their less active siblings [27]. Moreover, muscle-specific Fn14 knockout improved exercise capacity, vascular density, and resistance to muscle atrophy [26]. These findings suggest that Fn14, rather than TWEAK, is more responsive to exercise stimuli, with acute interventions inducing more pronounced changes. Simultaneously, both acute and aerobic exercise increase Fn14 mRNA and protein levels, with acute exercise exerting a more pronounced effect. However, exercise does not appear to significantly alter TWEAK expression. Additionally, Fn14 gene knockout enhances exercise capacity and resistance to muscle atrophy, but whether the transient post-exercise increase in Fn14 levels has similar effects remains unclear.

In this study, GSEA analysis showed that Fn14 is mainly enriched in apoptosis and cholesterol homeostasis pathways. Although Fn14-TWEAK interaction promotes apoptosis, the apoptosis pathway was suppressed after eight weeks of aerobic exercise. PPI network and GSEA analyses revealed an indirect link and co-enrichment of Fn14 and *Fos* in the apoptosis pathway, suggesting their potential joint role in apoptosis regulation. However, their interaction remains unclear, and further studies are needed to determine the physiological relevance of long-term aerobic exercise-induced Fn14 reduction and its relationship with *Fos*.

### 4.2. Fos as a Potential Mediator of Inflammation Alleviation and Regenerative Modulation in Skeletal Muscle Following Long-Term Aerobic Exercise

Fos is a member of the *Fos* gene family, located on chromosome 14. It is also known as the AP-1 transcription factor subunit or p55. Similar to *Egr1*, it is IEGs that play an important role in regulating gene expression in response to various stimuli such as stress, cytokines, growth factors, and infections. *Fos* family members can interact with the basic leucine zipper (bZIP) domain of Jun family proteins to form AP-1 complex [28,29]. AP-1 is a transcription factor complex composed of various family members, including the Jun family (c-Jun, JunB, JunD), Fos family (c-Fos, FosB, Fra-1/FosL1, Fra-2/FosL2), Maf family (c-Maf, MafA, MafB, MafF, MafG, MafK, Nrl), and Atf family (Atf, Atf2, Atf3/Lrf1, Atf4, Atf5, Atf6B, Atf7, BATF, BATF2, BATF3) [30,31]. These family members can combine to form either homodimers or heterodimers. However, due to the absence of the bZIP domain, members of the Fos family can only form heterodimers with other family proteins to exert their gene regulatory functions [29,31]. Under the influence of various stimuli such as growth factors, inflammatory cytokines, ultraviolet radiation, and bacterial or viral infections, AP-1 family members dimerize to form a complete AP-1 complex and translocate from the cytoplasm to the nucleus. The bZIP basic region within AP-1 can bind to AP-1 binding sites (5′-TGAG/CTCA-3′) located in the promoter or enhancer regions of target genes, thereby regulating the transcription of downstream target genes and modulating DNA transcription [29,32,33]. Additionally, the MAPK/ERK1/2 pathway can upregulate *Fos* expression, allowing it to participate in various physiological processes, such as cell proliferation, differentiation, stress responses, and neuronal activities [34]. However, excessive upregulation of *Fos* may lead to cell apoptosis and the development of cancer. Jee et al. [35] discovered a significant upregulation of *Fos* in cancer cells using RNA-seq technology. Moreover, by employing siRNA transfection techniques to knock down *Fos* expression in the CT26 cell line, they found that the downregulation of *Fos* reduced the proliferation of mouse colorectal cancer cells by approximately 20%, and survival analysis indicated that mice with low *Fos* expression exhibited better survival outcomes.

#### 4.2.1. Fos-Mediated Inflammatory Signaling and Its Modulation by Exercise

The expression level of *Fos* is closely related to aging and the tissue’s inflammatory response. The AP-1 complex, formed by *Fos* and Jun, is a central mediator of senescence-related inflammatory processes, while anti-aging regulators such as Sirt3 can suppress inflammation by inhibiting *Fos* transcription [36,37]. This suggests that sustained high expression of *Fos* is highly correlated with inflammation induced by aging. Upstream kinases like MLK3 have also been shown to activate *Fos* through phosphorylation, leading to the upregulation of pro-inflammatory cytokines such as COX-2, IL-6, and TNF-α [38]. Moreover, the activation of the NF-κB pathway can further modulate *Fos* expression. For instance, acute swimming exercise increases c-Fos expression in spinal neurons, an effect attenuated by NF-κB inhibitors. This study indicates that the NF-κB inflammatory pathway is involved in regulating c-Fos expression in spinal neurons, which is related to muscle delayed onset muscle soreness [39]. In skeletal muscle, inflammatory signaling involves multiple cascades (e.g., p38, ERK, JNK, and JAK), converging on transcription factors like NF-κB, AP-1 (Fos/Jun), and STATs, all of which promote the expression of inflammatory mediators [40,41]. Consistent with this, transcriptomic analyses have demonstrated that acute exercise activates the NF-κB pathway and triggers a robust inflammatory gene response in skeletal muscle [42], which indicates that acute exercise can induce skeletal muscle inflammation and activate a series of biological processes.

In this study, GSEA analysis showed that long-term aerobic exercise downregulated markers of the NF-κB signaling and inflammatory response pathways, indicating suppression of skeletal muscle inflammation. Notably, IEGs such as *Fos*, *Egr1*, and *Atf3*—which were significantly downregulated—were enriched in the NF-κB pathway, suggesting a potential link between IEGs suppression and inflammatory inhibition. Furthermore, ssGSEA immune infiltration analysis revealed that *Fos* expression was associated with Th1, Th2, and Treg cell infiltration, implying that long-term aerobic exercise may modulate T cell composition by repressing early gene expression. These findings suggest that *Fos* downregulation may help alleviate age-related skeletal muscle inflammation and improve muscle function and metabolism.

#### 4.2.2. Fos Regulates Skeletal Muscle Differentiation and Regeneration

In addition to modulating inflammatory signaling, *Fos* has a significant impact on the differentiation of skeletal muscle. Multiple studies have demonstrated that the downregulation of *Fos* is essential for muscle differentiation, as its sustained expression can inhibit myotube formation and suppress the expression of key muscle markers such as α-actin and myosin heavy chain [43]. The muscle-specific transcription factor MyoD promotes differentiation partly through the repression of c-Fos, and members of the AP-1 family (e.g., c-Fos, Fra-2, JunD) are known to regulate genes involved in myogenesis, such as *Mustn1* [44,45,46]. Moreover, *Fos* is responsive to mechanical stress; increased muscle tension due to contractions or stretching leads to elevated *Fos* expression, which has been observed in satellite cells during early stress responses and muscle regeneration. However, persistent *Fos* activation may disrupt chromatin architecture near myogenic genes and impair muscle formation [47,48,49]. These findings collectively suggest that the close regulation of *Fos* is essential for maintaining the balance between muscle growth and regeneration.

In this study, we found that, unlike acute exercise, aerobic exercise reverses the effect of acute exercise in upregulating *Fos*. GO biological process analysis revealed significant enrichment in muscle differentiation and development, involving genes such as *Fos*, *Egr1*, and *Atf3*, which are IEGs. Our analysis through single-cell transcriptomics showed that *Fos* and *Egr1* are primarily enriched in satellite cells, fibro/adipogenic progenitor cells, and myocytes, all of which are related to muscle regeneration and differentiation [50].

#### 4.2.3. Fos Expression in Response to Acute and Long-Term Exercise

Exercise exerts widespread regulatory effects on *Fos* expression across multiple tissues. Acute exercise has been shown to upregulate *Fos* in the liver, spinal cord, and brain, accompanied by increases in inflammatory cytokines and neuronal activation markers [39,51,52,53]. The expression of c-Fos in the hippocampus is influenced by both exercise intensity and duration—initially upregulated, but downregulated with long-term training [54]. Currently, research on the regulation of the *Fos* gene in skeletal muscle by exercise is limited and mainly focuses on phenotypic studies of *Fos*. Puntschart et al. [55] reported that acute exercise rapidly induces *Fos* mRNA and protein expression, which gradually decline within two hours. Zeng [56] observed that protein supplementation could attenuate the exercise-induced upregulation of *Fos*. Similarly, Nikolaidis et al. [57] and Rundqvist et al. [58] found that short-term high-intensity exercise elevated the expression of *Fos* and other IEGs. Chen et al. [40] demonstrated that exercise-induced *Fos* expression was further elevated in *Lkb1*-deficient mice, accompanied by exacerbated inflammation and muscle damage. In contrast, Jee et al. [35] reported that two weeks of high-intensity training led to the downregulation of *Fos* in the gastrocnemius muscle.

In this study, we analyzed datasets from both long-term and acute exercise using machine learning algorithms including Lasso, SVM-RFE, and Random Forest, along with PPI network and enrichment analysis. *Fos* was identified as a key gene involved in skeletal muscle growth, inflammation, and differentiation under long-term aerobic exercise. Additionally, similar to the brain’s hippocampal tissue, long-term aerobic exercise downregulates *Fos* expression in skeletal muscle, contrary to the results of short-term acute exercise interventions (Table 4). The upregulation of *Fos* in the early stages of acute exercise helps cells to respond to and adapt to environmental changes. In contrast, the downregulation of *Fos* indicates a reduction in the body’s inflammatory and injury responses following long-term aerobic exercise, suggesting that cells and tissues have adapted to continuous low- to moderate-intensity exercise. Consequently, the body has entered a stable physiological state, thereby reducing the need for frequent activation of genes associated with inflammation and injury repair, which lowers the sensitivity to chronic inflammation. Therefore, according to multi-omics data, *Fos* is highly likely to be a key factor inducing the heterogeneity of skeletal muscle differentiation in response to both acute and long-term exercise. These findings suggest that *Fos* is not only involved in the inflammatory response of muscle tissue, but also plays a crucial role in muscle differentiation, regeneration, and aging. In summary, this study reveals a distinct regulatory pattern of *Fos* expression under long-term aerobic exercise compared with acute exercise. The downregulation of *Fos* expression induced by long-term aerobic exercise may contribute to skeletal muscle regeneration, anti-aging, differentiation, and inflammation suppression.

Current research on the *Fos* and *Tnfrsf12a* genes in skeletal muscle mainly focuses on phenotypic changes, with limited insight into underlying regulatory mechanisms. Future studies should employ *Fos* and *Tnfrsf12a* specific inhibition and activation models to clarify its role in muscle differentiation and inflammation during both acute and long-term aerobic exercise. Given the divergent expression patterns of *Fos* under different exercise durations, further investigation is warranted into the upstream pathways regulating *Fos* and its potential links to muscle fiber type transformation or exercise adaptation. These findings may inform studies on athlete selection, performance, and skeletal muscle-related chronic diseases. Although machine learning was instrumental in identifying feature genes in this study, several limitations should be considered in future studies. First, model bias can arise from unbalanced or limited datasets, potentially leading to overfitting and reducing the generalizability of results. Second, feature selection methods such as Lasso, SVM-RFE, and Random Forest may yield different results depending on parameter tuning or data preprocessing, introducing variability and reducing reproducibility. Finally, machine learning results are correlative rather than causal, and thus require experimental validation to confirm biological causality.

## 5. Conclusions

Long-term aerobic exercise downregulates the expression of *Fos* and *Tnfrsf12a*, suggesting that this intervention may improve skeletal muscle function by inhibiting inflammation and promoting muscle differentiation and regeneration. Notably, the downregulation of *Fos* is more pronounced compared with *Tnfrsf12a*, and is closely associated with inflammatory responses and skeletal muscle regeneration. Simultaneously, *Fos* is a key distinguishing gene between long-term aerobic exercise and acute exercise. Overall, this study reveals the unique regulatory patterns of *Fos* and *Tnfrsf12a* following long-term aerobic exercise and provides new evidence supporting their roles as key molecular factors in skeletal muscle adaptation.

## Figures and Tables

**Table 1:** Data characteristics of the datasets involved in the expression matrix of this study.

Dataset ID	Tissue Type	Exercise Intensity and Duration	RNA-Seq Platform
GSE242354	Gastrocnemius	Incremental treadmill exercise at 70% VO_2max_ for 20–50 min daily, 8-week intervention	GPL25974
GSE242354	Vastus Lateralis		GPL25974
GSE222163	Gastrocnemius	Treadmill exercise at 12 m/min for 1 h daily, 8-week intervention	GPL23479
GSE198652	Skeletal Muscle	Progressive overload voluntary wheel running, 8-week intervention	GPL24247

**Table 2:** The nucleotide sequences of primers in RT-qPCR.

Primer Names	Nucleotide Sequences (5′-3′)
*Fos* Forward	CGGGTTTCAACGCCGACTA
*Fos* Reverse	TTGGCACTAGAGACGGACAGA
*Tm6sf1* Forward	AGTGCTGGCGTGTGAACTC
*Tm6sf1* Reverse	GTACAGTCCCAGATGTGCCTC
*Tnfrsf12a* Forward	GTGTTGGGATTCGGCTTGGT
*Tnfrsf12a* Reverse	GTCCATGCACTTGTCGAGGTC
*β-actin* Forward	CATGTACGTTGCTATCCAGGC
*β-actin* Reverse	CTCCTTAATGTCCGCACGAT

**Table 3:** Feature genes selected by three machine learning algorithms in the training set and their expression in the validation set GSE221210.

Genes’ Symbol	Log_2_FC		adj.P.Val		Consistency in Two Sets Training Set
	Training Set	Validation Set	Training Set	Validation Set	
*Hspa4l*	0.42419	0.13351	9.54 × 10^−12^	0.54348	Not consistent
*Atp1a1*	−0.39225	0.07806	4.02 × 10^−09^	0.75947	Not consistent
*Tm6sf1*	0.51454	0.51059	7.85 × 10^−09^	2.10 × 10^−03^	Consistent
*Tiam2*	−0.57921	−0.26645	2.96 × 10^−08^	0.47837	Not consistent
*Fos*	−0.93251	−1.40288	4.34 × 10^−07^	2.06 × 10^−13^	Consistent
*Socs2*	0.63069	0.08092	7.18 × 10^−06^	0.94107	Not consistent
*Tnfrsf12a*	−0.72025	−0.58546	1.32 × 10^−04^	0.12832	Consistent

**Table 4:** Literature review on the effects of different exercise modalities on *Fos* expression in skeletal muscle.

Author	Study Subjects	Comparison Method	Exercise Duration and Intensity	Intervention Period	Sample Type	Main Results of *Fos* Gene
Puntschart et al. [55]	5 untrained humans	Pre- and post-comparison	Single session of 30 min at individual anaerobic threshold intensity	Single session	Vastus lateralis	Increased *Fos* transcription levels and protein expression
Rundqvist et al. [58]	14 healthy individuals	Pre- and post-comparison	30 s per session at intensity of 0.075 kp/kg body weight	3 sessions	Quadriceps	Increased *Fos* transcription levels
Zeng et al. [56]	4 human skeletal muscle datasets from GEO	Pre- and post-comparison	Various exercise intensities	At least 2 weeks	Vastus lateralis	Increased *Fos* transcription levels
Nikolaidis et al. [57]	50 Wistar rats	Comparison with control	45 min per session at 20 m/min intensity	5 consecutive days	Extensor digitorum longus	Significantly increased *Fos* protein levels
Chen et al. [40]	12 skmLKB1-KO mice	Comparison with control	Single session of 61 min at 12 m/min, −17° slope	Single session	Quadriceps	Increased *Fos* transcription levels
Jee et al. [35]	40 male CDF1 mice	Comparison with control	45 min per session at 1 km/h intensity (90% of max heart rate)	19 days	Right gastrocnemius	Decreased *Fos* transcription levels

## Data Availability

The data presented in this study are available in the Gene Expression Omnibus (GEO) repository at https://www.ncbi.nlm.nih.gov/geo/ (accessed on 21 May 2024), under the reference numbers GSE198625, GSE242354, GSE222163, GSE221210 and GSE183288. These datasets were derived from publicly available resources, which can be accessed via the following URLs: GEO Database: https://www.ncbi.nlm.nih.gov/geo/ (accessed on 6 July 2024); GSE198625: https://www.ncbi.nlm.nih.gov/geo/query/acc.cgi?acc=GSE198625 (accessed on 21 September 2024); GSE242354: https://www.ncbi.nlm.nih.gov/geo/query/acc.cgi?acc=GSE242354 (accessed on 21 September 2024); GSE222163: https://www.ncbi.nlm.nih.gov/geo/query/acc.cgi?acc=GSE222163 (accessed on 22 September 2024); GSE221210: https://www.ncbi.nlm.nih.gov/geo/query/acc.cgi?acc=GSE221210 (accessed on 22 September 2024); GSE183288: https://www.ncbi.nlm.nih.gov/geo/query/acc.cgi?acc=GSE183288 (accessed on 24 September 2024); Please refer to the Supplementary Materials for further details on additional datasets and associated links.

## Supplementary Materials

The following supporting information can be downloaded at: https://zenodo.org/records/15383156 (accessed on 18 May 2025).
